# Weather Variables Associated with Spore Dispersal of *Lecanosticta acicola* Causing Pine Needle Blight in Northern Spain

**DOI:** 10.3390/plants10122788

**Published:** 2021-12-16

**Authors:** Nebai Mesanza, David García-García, Elena R. Raposo, Rosa Raposo, Maialen Iturbide, Mª Teresa Pascual, Iskander Barrena, Amaia Urkola, Nagore Berano, Aitor Sáez de Zerain, Eugenia Iturritxa

**Affiliations:** 1 Neiker BRTA, Instituto Vasco de Investigación y Desarrollo Agrario, 01192 Arkaute, Spain; aitorzerain41@gmail.com; 2 CIBERESP-Centro Nacional de Epidemiología, Instituto de Salud Carlos III, 28029 Madrid, Spain; david.garciag@externos.isciii.es; 3 The Centre for Linguistic Theory and Studies in Probability (CLASP), Göteborg University, 40530 Goteborg, Sweden; Elena.rraposo@gmail.com; 4 Intituto de Investigación Forestal, Instituto Nacional de Investigación y Tecnología Agraria (INIA-CIFOR-CSIC), 28040 Madrid, Spain; 5 Sustainable Forest Management Research Institute, University of Valladolid-INIA, 34004 Palencia, Spain; 6 Meteorology Group, Instituto de Física de Cantabria, CSIC-University of Cantabria, 39005 Santander, Spain; maibide@gmail.com; 7 Basalan, 48196 Lezama, Spain; ikerkuntza@basalan.eus (M.T.P.); elvira15@gmail.com (I.B.); 8 Laboratorio Agroambiental Fraisoro, Diputación de Gipuzkoa, Finca Fraisoro SN, 20159 Zizurkil, Spain; amaiaurkola@gipuzkoa.eus (A.U.); serologia22@gipuzkoa.eus (N.B.)

**Keywords:** *Lecanosticta acicola*, conidiospores, weather variables, generalized additive models

## Abstract

In the last decade, the impact of needle blight fungal pathogens on the health status of forests in northern Spain has marked a turning point in forest production systems based on *Pinus radiata* species. Dothistroma needle blight caused by *Dothistroma septosporum* and *D. pini*, and brown spot needle blight caused by *Lecanosticta acicola*, coexist in these ecosystems. There is a clear dominance of *L. acicola* with respect to the other two pathogens and evidence of sexual reproduction in the area. Understanding *L. acicola* spore dispersal dynamics within climatic determinants is necessary to establish more efficient management strategies to increase the sustainability of forest ecosystems. In this study, spore counts of 15 spore traps placed in *Pinus* ecosystems were recorded in 2019 and spore abundance dependency on weather data was analysed using generalised additive models. During the collection period, the model that best fit the number of trapped spores included the daily maximum temperature and daily cumulative precipitation, which was associated to higher spore counts. The presence of conidia was detected from January and maximum peaks of spore dispersal were generally observed from September to November.

## 1. Introduction

Needle blights are some of the most seriously damaging diseases affecting the health of *Pinus radiata* in Spain. The largest outbreak of needle diseases in the recorded history of *P. radiata* in northern Spain began in 2018 and has led to a significant modification of the landscape from a serious reconsideration of silviculture in the forestry sector. Needle blights identified in the study area include Dothistroma needle blight (DNB) caused by *Dothistroma septosporum* (Dorogin) Morelet and *D. pini* Hulbary, and brown spot needle blight caused by *Lecanosticta acicola* (Thümen) Sydow. The latter is by far the most frequent and abundant in *P. radiata* plantations [1]. In the last two decades, reports of *L. acicola* expansion in the northern hemisphere have increased, and also the number of host species and the climatic conditions in which this pathogen is detected [2]. Changes in the climate were associated with its spreading in the northeastern United States and Canada [3,4,5].

Conidia release of *L. acicola* mainly occurs by rain splash, a characteristic of fungi with a mucilaginous spore matrix [2]. Thus, conidiospore dispersal aligns with the rainfall season of the specific region being studied and depends on related factors such as rainfall occurrence and relative humidity [2,5]. Another variable that can directly or indirectly affect spore dispersal is temperature. The temperature range for conidia dispersion is −5.5 °C to 28 °C [2]. Temperatures above 30 °C negatively influence the germination, growth, and sporulation of *L. acicola* [5]. Conidia presence in forest ecosystems has been detected from spring to winter, with peaks in spore detection occurring from early summer to late autumn in one or two maximum time periods [5,6,7]. The capture of conidia is limited to short distances. For example, Wyka et al. [5] rarely detected spores at distances more than 3.05 m from the source tree, while, in another study, dispersion to adjacent trees resulted in the rapid spread of disease in forest stands [2]. Long-distance dispersal of *L. acicola* conidiospores has been linked to strong winds during rain, insects and especially silviculture practices involving the movement of infected plant material and tools [4,7]. Avoiding the new introduction of plant material is even more important because the increase in genetic diversity of the pathogen can enhance its potential aggressiveness or virulence [8].

*Lecanosticta acicola* ascospores are considered airborne and, thus, can be dispersed over long distances mainly by air currents [9]. However, their presence is less frequent. For instance, in Europe, although there is evidence indicating that sexual reproduction exists when both mating-type idiomorphs are present [2,10], the sexual stage was only recently confirmed [11]. 

Control of *L. acicola* is difficult because it can survive in both dead and living needles in forest ecosystems [9]. In addition, the infectious success of this pathogen increases due to a better adaptation to different temperature ranges [2] of southern and northern *L. acicola* lineages. Severe infection caused by *L. acicola* has a serious impact on growth and, in extreme cases, causes tree mortality [12,13]. Several measures have been suggested to minimise and prevent needle blight during plantation establishment and management. These include the use of healthy and good quality propagation material in areas far from infected pines [7,14,15] and the application of thinning treatments [16]. Thinning in winter was reportedly effective in reducing the severity of the disease in native stands of *P. strobus* in the United States [16]. However, local silvicultural management, including pruning and thinning, performed mainly in *P. radiata* plantations in the Atlantic area of Spain, did not result in the expected improvement [1]. In this region, 84% of the plantations are situated in an area with the highest climate risk factors for disease development, which might has impaired the effect of these management techniques [1]. The periods during which pruning activities of infected pines are executed are also relevant because conidiospores are discharged during rainy or wet periods and can adhere to the pruning saw blades, constituting a disease pathway from infected to healthy trees [7]. 

To minimise the infection of *L. acicola* through forest activities, it is important to understand the dynamics of spore dispersal and the favourable environmental conditions for the infection within a determined area. Activities that can reduce the disease impact may be implemented at times in which their effects could be more efficient against the disease.

The objective of this study was to monitor the production and dispersal of conidiospores of *L. acicola* in *P. radiata* ecosystems, representative of the Atlantic climate, with the aim of modeling spore abundance as influenced by the weather.

## 2. Materials and Methods

### 2.1. Spore Traps Location

**Figure 1:**
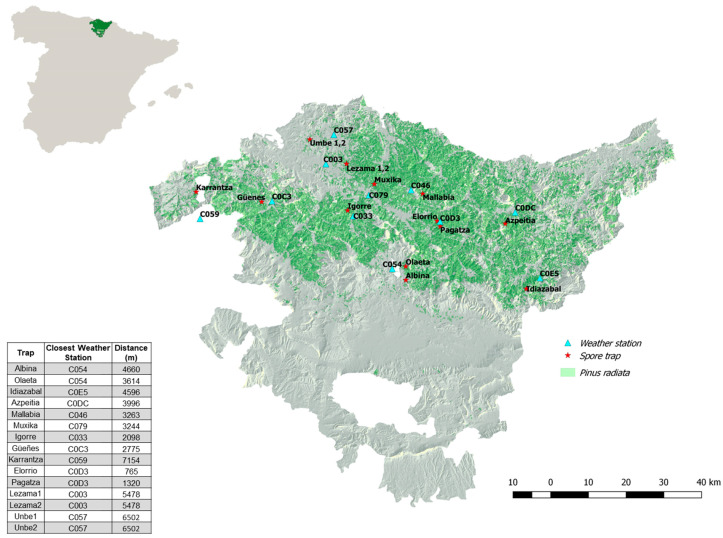
Location of spore traps (red) and weather stations (blue) in the Basque Country. Information about the distance between them is included in the bottom left of the image. The distribution of *Pinus radiata* appears in green on the map. Map with the location of the Basque Country in Spain is shown in the upper left corner of the figure.

A total of 15 spore traps were placed in *P. radiata* plantations in the Basque Country (Figure 1). The stands were selected because of their high levels of defoliation caused by *L. acicola* (confirmed by tree symptoms and identification of the pathogen by molecular methods and fungal morphology) [1] and because their locations covered different climatic regions. Levels of defoliation were quantified by the 5% intervals method [17]. 

Traps were placed in the interior of the plot, avoiding the edges at a distance from the closest trees of approximately 1.5 m. Traps were diagonally located in the transect following the distribution of *P. radiata* towards the coast. Two were located in the province of Araba, two in the province of Gipuzkoa, and 11 in the province of Bizkaia (Figure 1). The characteristics related to each trap location are listed in Table 1. The traps corresponding to the locations of Albina, Oleta, Lezama, Unbe, Pagatza and Elorrio were set on the sites on 7 January 2019. The traps corresponding to the locations of Mallabia, Muxika, Igorre, Güeñes and Karrantza were set on 31 January 2019, and those located on Idiazabal-Larraegi and Azpeitia-Igarate were set on 4 February 2019. 

### 2.2. Design and Measurements of Spore Traps

The passive impact spore traps were based on the design of Iturritxa and Ganley [18]. Four microscope slides were positioned vertically on an expanded polystyrene disk, approximately 6 cm thick and 9 cm in diameter, and covered by a Petri plate 9 cm in diameter. Four gaps were carved in the polystyrene base, thus that the slides formed a cross shape. To support the Petri plate, a hole was drilled in its centre, and a 9 cm nail was inserted. The nail point was affixed to the polystyrene disk. One side of each slide was covered with a thin layer of technical grade soft Vaseline (Panreac Applichem, Barcelona, Spain) before being placed in the base. Each trap was attached to the end of a 1.70 m post.

Microscope slides were collected approximately every 2 weeks, and spores were counted with a microscope using a 40× objective. Conidia were identified based on the morphological description of *L. acicola* described previously and were easily distinguished from other spores because of their slight crescent shape and olive colour [5,12]. The measured area on the slide was calculated by dividing the length of the measurement on the slide by the field of view (FOV). The FOV is the ratio of the microscope field number (22 mm) to the objective magnification [19]. The length of the measurement was set as the length of the cover slip. Once the area was determined, the number of spores per m^2^ was calculated and the spore concentration of the four slides was totalled. This measurement was normalised to the days elapsed since the change in the slides until their collection. Differences in the average number of spores trapped in different months were compared using the Kruskal–Wallis test in IBM SPSS (version 27.0.; IBM Corp. Armonk, NY) [20,21]. 

### 2.3. Meteorological Data

To determine the effects of environmental conditions on spore abundance, 10 variables were included for model parameterisation, which measured 5 different meteorological parameters. The parameters and corresponding variables were temperature (daily maximum, daily minimum, daily mean in °C), rainfall (daily cumulative precipitation in mm, number of days with >1 mm cumulative precipitation), irradiance (daily maximum and daily mean in W/m^2^), relative humidity (daily maximum and daily mean), and wind speed (daily average speed, in km/h).

Although historical weather reanalysis datasets with a fine geographical resolution exist, we chose to use observed meteorological data from the network of weather stations of Euskalmet, the Basque Country Public Meteorological Institute [22]. This was conducted in order to incorporate direct measurements and, thus, avoid potential confounding intermediate effects in the statistical models. Another reason for this is that the conclusions obtained from the models will be readily available for the surveillance teams and decision-makers on-site. Meteorological data were assigned to traps by selecting variables from the closest station and completing missing variables by proximity in cases where relevant variables were missing, up to a maximum distance of 10 km. After checking for erratic values and filtering, meteorological variables were calculated for the period of spore trapping. The supplied information had a 10-minute frequency that was aggregated accordingly to compute daily values, and then aggregated again to compute summary variables for the time periods between each spore count (sum of the number of days with >1 mm^3^ cumulative precipitation, mean for the rest of the variables). All extraction and transformation of the data were performed in R version 4.0.3 [23].

### 2.4. Statistical Analysis

We used a generalised additive model (GAM) [24] to analyse spore abundance dependency on weather data. GAMs assume a combination of linear and nonlinear (smooth) effects of the explanatory variables in the dependent variables. The nonlinear effects are smooth functions constructed as linear combinations of a fixed set of basis functions. A higher number of basis functions used in the construction of a smooth function accounts for the more complex effect of the corresponding variable. All statistical analyses were performed in R, version 4.0.3 using the ‘mgcv’ package [24].

A preliminary analysis revealed strong correlations among the meteorological variables that measured the same parameters. In particular, the variables within each group (temperature, rainfall, humidity and irradiance) had pair-to-pair correlations >0.8. To select the best variables within each group with these highly correlated variables, we fitted several GAMs and selected the best among them as follows (all models shared the same common structure):

*spore count* = *s* (*time*) + *re* (*trap*) + *temp* + *rainfall* + *humidity* + *irrad* + *wind*

where *s* (*time*) represents a smooth function of time that accounts for the temporal evolution of the response variable, and *re* (*trap*) models the location of each observation as a random effect. The remaining variables represent the linear effects of any of the variables measuring the corresponding parameter. That is, the *temp* can model daily maximum, mean, or minimum temperature; *rainfall* denotes daily cumulative precipitation or days with >1 mm; *humidity* represents the daily maximum or mean relative humidity; *irrad* denotes daily maximum or mean irradiance; and *wind* always corresponds to daily average wind speed. We considered each possible combination of variables to measure each of the parameters and fit 4 models for each combination. These 4 models have a different number of basis functions in the construction of the temporal component *s* (*time*) (k = 6, 8, 10, 12), and were included in the model selection process to seek an equilibrium between less precise and over-fitted models. We then chose the model with the lowest Akaike Information Criterion (AIC) score among all the fitted models (4 × 3 × 2 × 2 × 2 = 96 total) and inspected the meteorological variables used in this model, as well as those with the closest AIC scores.

As a check for the contribution of the weather variables to the estimation, we chose the statistically significant variables of the best model in the AIC score and fitted a model that only included these as linear covariates. We then compared its precision (% of deviance explained) to a null model with no weather covariates and to a full model, including the 10 weather variables under analysis.

We also tested the robustness of the best model upon changes in the input data in the following manner. We fit the same model to the dataset resulting from leaving out the measurements from one trap and compared the precision of this model to that of the original one (% of deviance explained) to search for a significant improvement or loss in precision. We repeated this process and removed further traps until the largest improvement in deviance explained by the removal of a single trap was <2%.

## 3. Results

### 3.1. Measures of Spore Dispersal

All 15 locations showed positive detection of *L. acicola*, although the number of captured spores varied significantly among locations and time periods. During the collection period, maximum peaks of spore presence were observed from September to November in 7 of the 15 traps (Unbe, Lezama, Igorre, Muxika, Elorrio, Mallabia and Olaeta). In most of them, a small increase in spore concentration occurred in May and July, except in Elorrio, where the peak detected in May was almost as high as that detected in September, and in Olaeta, which presented a high peak in early August. In two of the traps (Azpeitia and Idiazabal), although a high spore concentration was observed in the fall, the maximum values were detected from the end of May to the beginning of June. Three of the traps did not register spore concentration maximum periods as the others, with their peaks evident in February (Pagatza) or April (Güeñes and Karrantza). In these last three locations, only a small increase in spore concentration was observed during the fall. In Albina, the maximum spore concentration was detected in April, and high spore peaks appeared in February and November (Table 2). When comparing spore abundance measurements among all the locations, the maximum amounts were detected in the traps of Lezama 1 (1,446,791 spores/m^2^/day), Pagatza (582,592 spores/m^2^/day), Lezama 2 (578,716 spores/m^2^/day) and Unbe 1 (504,439 spores/m^2^/day). 

**Figure 2:**
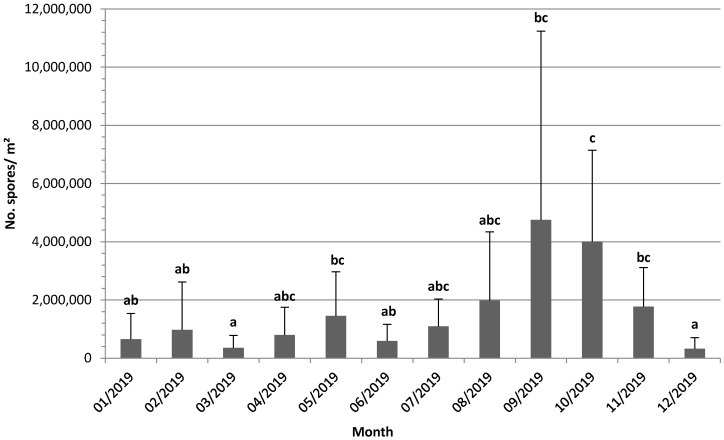
Mean of the number of spores per square meter per day in all trap locations during the assay period. Error bars indicate the standard error. The months marked with the same letters are not significantly different (*p* > 0.05).

In all traps, spores were detected from the first measurement in January or February, except in Karrantza and Güeñes, where they started at the end of February (in the third measurement). Spore counts were significantly affected by the month of the year, according to the Kruskal–Wallis test (H(11) = 35.7, *p* < 0.001) (Figure 2). The average number of spores trapped in October was significantly larger than those in the rest of the months. Although there were periods in which spore presence was unnoticeable in all the locations, the minimum values for all the traps collectively occurred in March and December (Figure 2).

### 3.2. Statistical Analysis

The model that best explained spore load included daily maximum temperature, daily cumulative precipitation, daily maximum relative humidity, daily mean irradiance and average wind speed as meteorological variables. Of these, only daily maximum temperature and cumulative precipitation were statistically significant (Table 3).

Moreover, while the number of basis functions and the variables measuring relative humidity and irradiance varied among the best models, maximum temperature and cumulative precipitation consistently appeared in the top fitted models and showed a statistically significant and positive effect on the spore count. In particular, when sorting the models based on differences in the AIC score with the best model (∆*i*) and selecting those with ∆*i* < 2 (the usual rule of thumb for models with substantial support [25]), the daily maximum temperature and daily cumulative precipitation were included in all of these models (Table 4).

The model including only daily maximum temperature and cumulative precipitation as meteorological covariates yielded a 31.9% of explained deviance, with both variables being statistically significant (coefficients 76,027 and 55,281, respectively). The full model that included the 10 available variables showed a 1.4% improvement in deviance explained, in contrast to the 18.1% decrease yielded by the null without meteorological covariates.

When leaving out one of the traps from the dataset, there was an average change of 0.9% in the deviance explained in the model selected in the fitting process. Leaving out the measurements from a particular trap (Pagatza) increased the precision of the model to a 36.6% deviance explained. Leaving out the data from an additional trap (Lezama 1) yielded a further increase to 42.1% in deviance explained (Table 3). 

In view of the above results, we summarise the final model of our analysis in Table 5. This includes daily maximum temperature and cumulative precipitation as linear covariates (as these were the most, and only, significant variables for the estimation of spore load in the previous models) and fits them to data from all the traps, except Pagatza and Lezama 1 (as these seem to decrease the precision of the estimates). This model represents the most precise approximation of the effect of weather covariates on the number of spores. Thus, it is the one that should be used for surveillance and predictions upon further development and validation with future data collection.

## 4. Discussion

In this study, we modeled the dependency of spore abundance on weather data, as analysed by GAMs, based on the dispersal patterns of conidiospores of *L. acicola* in *P. radiata* ecosystems that are representative of the Atlantic climate during 2019.

Our analysis suggests that both high temperatures and precipitation contribute significantly to the appearance of *L. acicola* spores. More precisely, the maximum temperature and cumulative daily precipitation seem to be the best available indicators for an increase in spore count. Indeed, these variables provided a notable improvement to our statistical model. Adding more variables, up to a total of 10, only slightly changed the precision of the estimates. While the overall precision of the statistical model can still be improved (see below), we found a robust behaviour when restricting its input data (<1% average change in precision when leaving out measurements from one trap).

The observations at two particular traps, Pagatza and Lezama 1 seemed to have a confounding effect on the model, as removing them from the fitting process significantly improved the model’s precision. This could be due to a global limitation of our analysis, which is the fact that the trap locations and their associated weather stations do not coincide. We addressed this constraint by incorporating the trap location as a random effect in the model and by using the robustness controls included in the model fitting and selection process (see Methods). Nevertheless, the large distances between traps and stations (average distance to closest station 3.88 km, distance from Lezama 1 to closest station 5.47 km) and the local geography (weather station closest to Pagatza sits beside a large body of water) may still restrict the scope of our approach. In addition, the lack of precision for Lezama 1 could be due to the high number of spores, not because it is high per se, but because of the lack of more traps for these characteristics, the model does not achieve statistical solidity for these ranges of values.

We arrived at a ‘final’ model, incorporating the evidence obtained in our model selection process, as summarised in Table 3. While it provides our best guess for the effect of the available meteorological data on the spore count at the selected locations, it should be understood as an estimation tool for the moment. Another relevant constraint in our analysis is that the time series of spore counts consists of either 24 or 25 observations at each of the traps, a relatively small number that may prevent statistically robust conclusions from being drawn from the data. We hope to incorporate further measurements to corroborate our findings in future work and to develop a reliable predictive model based on the results of this investigation. A reliable prediction requires model validation to ensure a degree of agreement between the output of the model and any new record.

Conidiospore presence was detected in all traps from January or February to mid-November (Unbe 2) or December. In contrast, Wyka et al. [5] did not capture spores until the end of May in traps located in Maine. In Wisconsin and Minnesota, Skilling and Nicholls [7] did not observe conidiospores from December to the end of April. In the Shimane Prefecture (Japan), Suto [6] detected conidiospores from late March to late December. *Dothistroma septosporum* causes DNB and has similar symptomatology and spreading mechanisms to *L. acicola*. Dvorak et al. [26] detected the start of *D. septosporum* sporulation in April and May, which finished at the end of October in South Moravia (Czech Republic). 

During the collection period, the general pattern of spore dispersal peaked from September to November, with a small increase in spore concentration in May and July. There are a few exceptions where this second peak was almost as high as that detected in September (Elorrio, Azpeitia and Idiazabal) or in early August (Olaeta). Three locations did not register maximum spore amounts at those times, except in February (Pagatza) or April (Albina, Güeñes and Karrantza). In other studies, maximum peaks were detected in June and July [4,7]. These differences in spore dispersal starting points and maximum spore concentration peaks may be related to local precipitation and temperature patterns that are different in our study area. Additionally, different strains of the pathogen can exhibit a certain degree of adaptability to local conditions [10]. In our study, the southern lineage of *L. acicola* is predominant and southern isolates have been reported to be more virulent to *Pinus* spp. than northern ones, except for *P. sylvestris*. Southern isolates have also been reported to be better adapted to higher temperatures [27,28]. Thus, the importance of local studies in the proper management of forest diseases is of great importance.

This study focused on the quantification of *L. acicola* conidiospores, although the presence of the sexual form was recently confirmed in the area, the infrequent ascocarp detection and the nature of the traps used may be the reason for the absence of ascospores during the screening. Passive impact spore traps often result in ineffective capture of airborne sexual spores of *L. acicola*, even in regions where their presence is expected [4]. The ratio of the presence of the sexual form to the asexual form was greatly inferior in needle samples collected in the area of study [11].

This study will be extended for several years, giving us the opportunity to apply predictive models and evaluate how this spread behaviour could change within this time. Because Spain is one of the regions most vulnerable to the direct impacts of climate change, it is experiencing rises in temperature, floods and droughts. Spore production and discharge seem to be favoured by warm conditions and rainy or wet periods, which may have implications for future climate scenarios. In northern Spain, spring temperatures have increased over the past decades [28]. According to the Intergovernmental Panel on Climate Change, climate change could increase average temperatures, by 2–4 °C, in Europe over the next 50 years and cause considerable changes in regional and seasonal patterns of precipitation. In the study area, a 15% reduction in annual precipitation is expected by 2100 and a rise in atmospheric temperature by 1.5 °C to 5 °C [29]. Current projections from regional climate models indicate the warming of surface air over the Basque Country. In particular, heatwave episodes will increase in duration, and the 90th percentile of daily maximum temperature is expected to increase during summer by 3 ± 0.9 °C [29]. Monitoring the spore-spreading capacity of forest diseases could be a good indicator for studies of climate change impact and for evaluating the adaptation capacity of forest fungal species and hosts to new climate scenarios. Spore traps, in addition to being an efficient method to carry out this type of study, are easy to build and inexpensive, which gives them greater versatility to solve disease management issues [5].

The generation of disease databases at local and global scales in regional climate change scenarios is a fundamental starting point in assessing impacts, vulnerability and future needs with respect to adaptation to these outbreaks of forest disease [5].

## 5. Conclusions

Knowledge of pathogens dispersal dynamics related to climate variables is important when forest management strategies need to be implemented. In this study, the abundance of *L. acicola* conidiospores was measured in the Spanish Atlantic region during 2019, and the dependency of observations to weather determinants was analysed by GMAs. The analysis suggests that the maximum daily temperature and cumulative daily precipitation are the best available indicators for an increase in spore captures. These variables provided a notable improvement to our statistical model. In the future, further spore count measurements for different years will be included to develop a reliable predictive model. 

## Figures and Tables

**Table 1:** Locations of spore traps and stand characteristics.

Trap ID	Province	X Coordinates	Y Coordinates	Orientation	Slope (%)	Age	Defoliation Level of Site (%)
Albina	Araba	531,468	4,762,368	Southeast	5 to 10	13	25
Oleta	Araba	531,448	4,765,973	Southwest	20 to 30	9	30
Idiazabal Larraegi	Gipuzkoa	563,439	4,760,042	Southwest	30 to 50	4	30
Azpeitia Igarate	Gipuzkoa	557,840	4,777,305	Northwest	30 to 50	9	70
Mallabia	Bizkaia	535,952	4,785,234	Northeast	20 to 30	<15	>30
Muxika	Bizkaia	523,110	4,787,806	Northeast	30 to 50	<15	>30
Igorre	Bizkaia	516,044	4,780,811	South	30 to 50	<15	>30
Güeñes	Bizkaia	493,228	4,783,097	Northeast	50 to 100	<15	>30
Karrantza	Bizkaia	475,875	4,785,737	West	10 to 20	<15	>30
Elorrio	Bizkaia	539,801	4,778,037	Northwest	10 to 20	4	50
Pagatza	Gipuzkoa	540,597	4,776,579	North	10 to 20	12	55
Lezama1	Bizkaia	515,746	4,793,192	South	20 to 30	5	55
Lezama2	Bizkaia	515,746	4,793,192	South	20 to 30	5	55
Umbe1	Bizkaia	506,024	4,799,627	North	5 to 10	14	50
Umbe2	Bizkaia	506,024	4,799,627	North	5 to 10	14	50

**Table 2:** Number of spores per square meter per day for each trap in the assay period. Maximum spore values for each location are represented by bold letters. ND: no data.

	Idiazabal	Azpeitia	Karrantza	Güeñes	Igorre	Muxika	Mallabia	Unbe1	Unbe2	Lezama1	Lezama2	Elorrio	Pagatza	Olaeta	Albina
07/01/2019	ND	ND	ND	ND	ND	ND	ND	50,295	6035	ND	ND	18,106	42,247	4024	2012
21/01/2019	ND	ND	ND	ND	ND	ND	ND	116,683	54,318	261,532	74,436	114,672	86,507	21,906	0
04/02/2019	ND	ND	0	0	18,505	14,235	39,858	26,746	10,698	77,564	40,119	18,722	2675	21,906	0
18/02/2019	6404	6404	0	0	0	0	0	0	0	35,524	0	0	**582,592**	19,824	88,105
04/03/2019	0	0	2496	0	17,474	7489	24,963	28,419	7105	10,657	17,762	28,419	3552	7105	0
18/03/2019	1949	1949	23,793	1322	7931	10,574	15,862	17,762	7105	0	0	0	0	0	0
01/04/2019	8966	0	7931	1322	5287	6609	6609	202,020	15,151	75,757	35,354	10,101	0	0	0
15/04/2019	0	25,617	**52,459**	**34,973**	31,475	13,989	13,989	202,020	15,152	75,758	35,354	10,101	0	0	**102,973**
29/04/2019	25,617	6404	3264	3264	11,424	8160	8160	9946	2486	134,280	14,920	37,300	12,433	12,433	7460
13/05/2019	2989	13,185	5649	0	7533	5649	1883	31,971	3552	142,096	120,781	284,191	28,419	0	0
27/05/2019	44,830	**110,036**	22,732	3497	110,164	66,448	57,705	14,210	63,943	92,362	81,705	138,543	56,838	31,971	0
10/06/2019	**50,104**	5274	5649	0	28,247	5649	7533	29,840	6631	46,418	72,942	66,311	0	46,181	0
24/06/2019	16,302	0	0	0	36,721	40,219	0	0	11,477	3826	7651.	0	0	92,362	3552
08/07/2019	0	0	4080	0	134,645	96,564	28,561	0	7105	39,076	85,257	3552	49,733	23,272	18,864
22/07/2019	3202	0	4080	0	134,645	96,564	28,561	53,049	33,156	102,782	62,996	62,996	43,102	23,272	18,864
05/08/2019	3202	0	0	0	54,837	75,401	67,567	9947	4973	39,787	34,813	74,600	0	169,094	29,840
19/08/2019	0	0	0	0	54,837	75,401	67,567	46,807	4973	32,180	1755	32,180	1170	2925	2925
02/09/2019	10,345	0	1749	0	117,159	138,142	96,175	144,679	**226,061**	**1,446,791**	**578,716**	149,200	9042	60,391	10,657
16/09/2019	0	0	0	0	132,896	78,689	36,721	418,346	198,934	854,245	424,197	**359,836**	17,553	7105	0
30/09/2019	0	0	0	0	**342,733**	35,780	11,299	294,848	63,943	291,296	209,591	191,829	60,391	**177,619**	10,657
14/10/2019	35,864	65,750	0	0	172,998	127,301	**200,744**	195,381	39,076	255,772	209,591	269,982	14,210	134,991	40,260
28/10/2019	32,021	64,754	0	1748	125,902	**253,552**	103,169	**504,439**	195,381	319,715	298,401	127,886	14,210	134,991	40,260
11/11/2019	22,415	84,678	0	0	131,820	86,625	122,405	165,778	175,725	62,996	16,578	179,040	3316	71,048	60,391
25/11/2019	6897	32,875	1632	0	29,377	44,066	26,113	0	0	95,914	49,733	110,124	7105	29,840	6631
09/12/2019	0	4981	0	0	5246	13,989	1749	8913	0	41,592	77,243	8913	0	17,825	0
23/12/2019	4483	16,302	0	1632	27,745	8160	37,537	17,361	0	14,205	6313	17,361	1578	2185	0

**Table 3:** Summary of the linear effects of the meteorological variables in the best model in the Akaike Information Criterion (AIC) score when fitting data from all the traps, and in the same model fitted to data from all traps except those of Pagatza and Lezama 1.

	Data from All Traps		Leaving out Pagatza and Lezama 1	
**Variable**	**Coefficient**	**Std. Error**	**Coefficient**	**Std. Error**
Daily maximum temperature *	78,002	38,497	83,075	27,376
Daily cumulative precipitation *	47,580	26,445	44,785	19,572
Daily maximum relative humidity	12,280	19,662	10,527	13,340
Daily mean irradiance	−2106	2386	−1811	1650
Daily average wind speed	−41,958	100,710	−11,860	74,291

Statistically significant variables (with a significance value of 0.1) are marked with an asterisk (*).

**Table 4:** Weather variables and number of basis functions of the top 8 fitted models, ranked by ascending difference in AIC score with the best model ($\Delta_{i}$). *k* denotes the number of basis functions used in the construction of the smooth temporal component.

Model	Temp	Rainfull	Humidity	Irrad	Wind	*k*	Δ*i*
1	Daily maximum *	Cumulative precipitation *	Daily maximum	Daily mean	Average speed	8	0
2	Daily maximum *	Cumulative precipitation *	Daily mean	Daily mean	Average speed	8	0.683
3	Daily maximum *	Cumulative precipitation *	Daily maximum	Daily maximum	Average speed	8	0.725
4	Daily maximum *	Cumulative precipitation *	Daily maximum	Daily mean	Average speed	10	1.163
5	Daily maximum *	Cumulative precipitation *	Daily maximum	Daily mean	Average speed	12	1.373
6	Daily maximum *	Cumulative precipitation *	Daily mean	Daily maximum	Average speed	8	1.41
7	Daily maximum *	Cumulative precipitation *	Daily mean	Daily mean	Average speed	10	1.795
8	Daily maximum *	Cumulative precipitation *	Daily maximum	Daily maximum	Average speed	10	1.897

Statistically significant variables are marked with an asterisk (*).

**Table 5:** Summary of the final model of the analysis.

Final Model	Deviance Explained = 41.5%	k = 8 Basis Functions
Variable	Coefficient	Std. error
Daily maximum temperature *	77,652	26,153
Cumulative precipitation *	50,438	18,987

Statistically significant variables are marked with an asterisk (*).

## Data Availability

Data sharing is not applicable to this article.
